# Antipsychotic-Induced Parkinsonism With Concurrent Benztropine-Associated Anticholinergic Effects in a Community Emergency Department Setting: A Case Report

**DOI:** 10.7759/cureus.116368

**Published:** 2026-09-16

**Authors:** Colin Barnett, Manoj Sreedharan

**Affiliations:** 1 College of Osteopathic Medicine, Des Moines University, West Des Moines, USA; 2 Emergency Medicine, MercyOne Clinton Medical Center, Clinton, USA

**Keywords:** amantadine, anticholinergic toxicity, antipsychotic-induced parkinsonism, benztropine, community emergency department, extrapyramidal symptoms (eps), neuroleptic malignant syndrome (nms), paliperidone, schizophrenia, second-generation antipsychotics

## Abstract

Antipsychotic-induced parkinsonism (AIP) is a neurologic side effect of first- and second-generation antipsychotics. We present a case of AIP associated with paliperidone with findings concerning for concurrent anticholinergic side effects as a consequence of AIP treatment in a young patient presenting to a community emergency department. Temporarily withdrawing the offending dopamine receptor-blocking antipsychotic medication as well as implementing an adjunct therapy such as benztropine may alleviate the acute presentation of AIP. However, concurrent anticholinergic adverse effects or toxicity may occur because of the antimuscarinic properties of benztropine. In patients in whom anticholinergic toxicity or benztropine-related anticholinergic side effects are a concern, amantadine may be considered as an alternative treatment for AIP. This case report highlights the diagnostic complexity of AIP with suspected benztropine-induced anticholinergic effects, discusses management considerations of this complex presentation, and aims to guide proper treatment in community emergency department settings.

## Introduction

Antipsychotic-induced parkinsonism (AIP) is a well-documented and dose-dependent neurologic side effect of first- and second-generation antipsychotics, which are used for management of schizophrenia and other psychotic disorders [1,2]. Extrapyramidal side effects exist on a spectrum, such as parkinsonism, dystonia, tardive dyskinesia, and akathisia [1]. AIP is a manifestation of a clinical spectrum of extrapyramidal symptoms (EPS) associated with dopamine (D2) receptor blockade in the nigrostriatal pathway [3].

AIP is most commonly associated with first-generation antipsychotics such as haloperidol or fluphenazine but may also be less commonly induced by second-generation antipsychotics such as paliperidone [4]. In particular, paliperidone long-acting injectables (LAI) have been a mainstay treatment modality for schizophrenia, but may lead to EPS consequences, which may develop in as soon as a matter of days [5]. 

Common medications used to counteract such unwanted side effects from antipsychotic drugs include M1 receptor antagonists (anticholinergics) such as benztropine. Although benztropine is effective at alleviating EPS, there is a risk of anticholinergic toxidrome, characterized by flushing, anhidrosis, mydriasis, hyperthermia, dry mucous membranes, and even delirium [6]. 

We present a case of AIP complicated by concurrent anticholinergic side effects as a consequence of AIP treatment in a young patient presenting to a community emergency department with vague neurologic symptoms. This case highlights the importance of early recognition of signs and prompt treatment of AIP, as well as creating a proper differential diagnosis of similar conditions.

## Case presentation

Methods

This case was identified during routine clinical care in a community/rural emergency department in the Midwest. Clinical information was obtained through direct observation of patient care and retrospective review of the patient’s electronic medical record, including nursing notes, physician documentation, laboratory studies, imaging results, medication records, and past medical history. Additional details pertinent to the case were gathered through direct history and physical examination with the patient and family members.

Diagnostic evaluation and management were carried out as part of standard clinical care in an emergency department. Diagnostic evaluation was performed to evaluate potential traumatic, neurologic, metabolic, and toxicologic causes. All patient information was de-identified in accordance with institutional privacy guidelines. This case report was determined to be exempt from institutional review board because it involves a single de-identified patient without experimental intervention.

Results

The patient is a 24-year-old white male with a past medical history significant for schizophrenia who presented to the local community emergency department on day 0 for a recent fall and an increased unsteady gait. Upon eliciting a detailed history, it was established that the patient had been treated by psychiatry at another facility for schizophrenia on long-acting paliperidone injections (234 mg every 30 days), with his last dose five days prior to emergency department presentation. Additionally, benztropine (1 mg twice daily (BID) as needed) had been prescribed by his psychiatrist four days prior to ED presentation as needed for paliperidone side effects. His most recent dose was day one prior to presentation. 

Physical exam was positive for tachycardia, drooling, shuffling gait, intention tremors, cogwheel rigidity, pill-rolling tremor, occasional restlessness, diffuse muscle pains, increased weakness, dyskinesia, flat affect, and slowed behavior, with agitation and delirium. The patient was negative for dry mouth, sweating, and constipation. Over the course of the next few hours, the patient developed urinary retention, hypertension, and tachycardia. The patient's vitals upon admission were a maximum temperature of 36.9 C, blood pressure of 130/90, heart rate of 105, respiratory rate of 19, and oxygen saturation of 97% on room air. The patient was agitated and delirious. Pupils were 2 mm and sluggish, bilaterally. The remainder of the physical and neurologic exam was unremarkable. Further laboratory studies revealed an elevated WBC count (12.7 K/mcL) with neutrophilia (82.5 %), as well as elevated ketones (20 mg/dL), RBCs (0-2/high-power field (HPF)), and hyaline casts (0-2/low-power field (LPF)) with small mucus found upon urinalysis. Urine drug screen further showed presumptive positive benzodiazepines. The remainder of the laboratory studies including complete blood count (CBC), comprehensive metabolic panel (CMP), urine drug screen, urinalysis, microbiology, thyroid studies, ethanol level, salicylate level, acetaminophen level, and creatine kinase (CK) were unremarkable (Table 1). No follow-up CK laboratory studies were done during the remainder of the ED admission.

**Table 1:** Laboratory workup CBC: complete blood count; WBC: white blood cells; RBC: red blood cells; MCV: mean corpuscular volume; MCH: mean corpuscular hemoglobin; MCHC: mean corpuscular hemoglobin concentration; RDW: red cell distribution width; MPV: mean platelet volume; CMP: comprehensive metabolic panel; BUN: blood urea nitrogen; Cr: creatinine; eGFR: estimated glomerular filtration rate; AST: aspartate aminotransferase; ALT: alanine aminotransferase; TSH: thyroid stimulating hormone; T4: thyroxine; CK: creatine kinase; THC: tetrahydrocannabinol; PCP: phencyclidine; HPF: high-power field; LPF: low-power field; SARS-CoV-2: severe acute respiratory syndrome coronavirus 2

Category	Lab	Value	Reference Range
CBC	WBC	12.7 K/mcL	4.8 - 10.8 K/mcL
	RBC	5.19 M/mcL	4.50 - 6.00 M/mcL
	Hemoglobin	15.6 g/dL	13.5 - 18.0 g/dL
	Hematocrit	0.454	39.7 - 49.0 %
	MCV	87.3 FL	80.0 - 100.0 FL
	MCH	30.0 pcg	25.4 - 34.6 pcg
	MCHC	34.3 g/dL	31.0 - 36.0 g/dL
	RDW	0.132	11.5 - 14.5 %
	Platelets	276 K/mcL	150-450 K/mcL
	MPV	6.6 FL	6.6 - 10.4 FL
	Neutrophils	0.825	43.2 - 74.2 %
	Lymphocytes	0.09	15.8 - 46.8 %
	Monocytes	0.076	4.3 - 12.0 %
	Eosinophils	0.005	0.0 - 4.8%
	Basophils	0.004	0.3 - 1.5%
	Absolute Neutrophils	10.5	1.80 - 7.80 K/mcL
	Absolute Lymphocytes	1.1	1.00 - 3.00 K/mcL
	Absolute Monocytes	1	0.30 - 1.00 K/mcL
	Absolute Eosinophils	0.1	0.00 - 0.50 K/mcL
	Absolute Basophils	0	0.00 - 0.10 K/mcL
CMP	Sodium	137 mmol/L	134-150 mmol/L
	Potassium	4.0 mmol/L	3.3 - 5.2 mmol/L
	Chloride	102 mmol/L	98 - 111 mmol/L
	CO2	25 mmol/L	22 - 32 mmol/L
	Anion Gap	10	46219
	Glucose	85 mg/dL	70 - 100 mg/dL
	BUN	7 mg/dL	6 - 20 mg/dL
	Creatinine	1.00 mg/dL	0.70 - 1.20 mg/dL
	eGFR	108 mL/min/1.73m2	>= 60 mL/min/1.73m2
	BUN/Cr Ratio	7	9.3 - 32.1
	Calcium	9.8 mg/dL	8.6 - 10.3 mg/dL
	AST	17 units/L	15-41 units/L
	ALT	16 units/L	17-63 units/L
	Alkaline Phosphatase	63 units/L	32 - 91 units/L
	Total Protein	7.6 g/dL	6.1 - 7.9 g/dL
	Albumin	4.7 g/dL	3.5 - 4.8 g/dL
	Total Bilirubin	1.4 mg/dL	0.0 - 1.5 mg/dL
Other	Acetaminophen Level	<1.0 mcg/mL	10.0 - 30.0 mcg/mL
	Salicylate Level	<1.5 mg/dL	0.0 - 30.0 mg/dL
	Ethanol Level	<10 mg/dL	<= 10 mg/dL
	TSH	3.7 mclU/mL	0.30 - 5.60 mclU/mL
	Free T4	1.02 ng/dL	0.71 - 1.85 ng/dL
	CK (Total)	392 units/L	49 - 397 units/L
Urine Drug Screen	Amphetamines	Negative	Negative
	Barbiturates	Negative	Negative
	Benzodiazepines	Presumptive Positive	Negative
	Opiates	Negative	Negative
	Cocaine	Negative	Negative
	THC	Negative	Negative
	PCP	Negative	Negative
	Methadone	Negative	Negative
	Fentanyl	Negative	Negative
	Urine Creatinine	161.9 mg/dL	mg/dL
Urinalysis	Color	Yellow	Straw, Yellow
	Clarity	Clear	Clear
	pH, Urine	6.0 pH	5.0 - 8.0 pH
	Specific Gravity	1.014	1.003-1.030
	Glucose	Negative	Negative
	Protein	Negative	Negative
	Blood	Negative	Negative
	Leukocyte Esterase	Negative	Negative
	Nitrite	Negative	Negative
	Ketones	20 mg/dL	Negative
	Urobilinogen	Normal	Normal
	Bilirubin	Negative	Negative
	WBC	None Seen	None Seen
	RBC	0-2 /HPF	None Seen/HPF
	Squamous Epithelial Cells	None Seen	None Seen
	Bacteria	None Seen	None Seen
	Mucus	Small	None/HPF
	Hyaline Casts	0-2 /LPF	None Seen/LPF
Microbiology	Group A Strep	Not Detected	Not Detected
	Respiratory Viral Panel	Not Detected	Not Detected
	SARS-CoV-2	Not Detected	Not Detected

The patient received continuous monitoring and supportive care (sodium chloride 0.9% boluses). Both propranolol (10 mg BID) and diazepam (10 mg BID) were initiated upon ED presentation. The patient showed moderate improvement in mental status and parkinsonian symptoms on day 1 after overnight monitoring in the emergency department. Benztropine and paliperidone were withheld during the hospital course. Amantadine was then administered on day 2 as a replacement therapy for benztropine to manage the Parkinson-like side effects, and the patient was transferred to a nearby tertiary care hospital for further workup and management with an inpatient service with psychiatry and neurology consulting (Table 1).

**Table 2:** A patient with a history of schizophrenia, recently treated with long-acting paliperidone for schizophrenia, presented to the emergency department with findings consistent with neuroleptic-induced parkinsonism, subsequently treated with benztropine and quickly developed anticholinergic side effects. Both medications were discontinued, amantadine was initiated, and the patient was transferred to a tertiary care center with psychiatry and neurology consultation. LAI: long-acting injectable; ED: emergency department; BID: twice a day; PRN: as needed; IV: intravenous

Clinical Stage	Key findings/Events	Management/Outcome	
Paliperidone LAI therapy	Monthly paliperidone LAI (234 mg) for schizophrenia diagnosed 10 months ago; most recent dose 5 days prior to ED presentation.		
Benztropine initiation and dose escalation	Benztropine prescribed 4 days before ED presentation for extrapyramidal symptoms (1 mg BID PRN). Most recent dose 1 day before ED presentation.		
Emergency department presentation (Day 0)	Flat affect, weakness, shakiness, slowed responses, worsening gait instability, fall, and eventual inability to walk.	Cardiac monitoring started. Laboratory workup initiated.	
Evaluation and treatment (Day 0)	ED assessment favored neuroleptic-induced parkinsonism with anticholinergic effects worsened by benztropine.	Initiated supportive care. Treated with diazepam 10 mg PRN, propranolol 10 mg BID, and IV fluids.	
ED observation (Day 1)	Significant initial improvement in blood pressure and mental status, but persistent gait instability and recurrent tachycardia and urinary retention. Foley catheter required replacement. Output of >600 mL.	Continued supportive care and observation.	
Transfer to tertiary care (Day 2)	Started amantadine (100 mg BID). Persistent/recurrent symptoms despite partial improvement.	Transferred to tertiary care hospital for continued supportive care and neurologic and psychiatric evaluation.	

## Discussion

This case highlights the importance of early recognition of signs and prompt treatment of AIP, an underrecognized scenario of generalized weakness, rigidity, flat mood and affect, and pill-rolling tremor. This patient exhibited vague neurologic symptoms over the course of several weeks, which significantly worsened, requiring prompt emergency department evaluation and intervention with benztropine to treat AIP. 

Broad differential diagnoses should be made in cases such as this with patients who present with vague movement disorders in the context of antipsychotic treatment. These possibilities include catatonia, functional gait disorder, and so forth. It should also be noted that EPS exists on a spectrum such as parkinsonism, dystonia, tardive dyskinesia (TD), and akathisia [1]. Given the overall clinical picture, this case most directly points to parkinsonism, specifically. The cogwheel rigidity, shuffling gait, tremor, etc. support this diagnosis, along with the time frame of insidious onset over a few weeks, with substantial worsening over a few days, leading to emergency department presentation. Akathisia is less likely due to the fact that the patient was slow with a shuffling gait, rather than restless. Moreover, tardive dyskinesia would have manifested over weeks to months, which does not match the timeline of this acute presentation. Acute dystonia was further down on the differential diagnosis in this case because although some tremors could have been mistaken for spasms, this patient had a hypokinetic syndrome of rigidity and tremor, along with other signs more consistent with AIP, specifically. Other diagnoses such as catatonia were ruled out in this case, due to the lack of behavioral-volitional signs that anchor catatonia as a primary diagnosis. Additionally, the patient did not likely have a functional gait disorder because of an organic cause attributable to antipsychotic treatment. Nonetheless, creating a unifying diagnosis for this case was difficult due to a myriad of overlapping symptoms and determining temporal and causal relationships between the clinical findings. The final diagnosis from the emergency department was not absolutely definitive, rather a working diagnosis supported by clinical judgement. Similar cases to this one should be carefully evaluated to distinguish similar, but distinct diagnoses. 

Furthermore, this patient's presentation was made difficult by suspected anticholinergic effects due to the addition of benztropine in an attempt to treat the EPS. This patient exhibited autonomic instability such as tachycardia, hypertension, altered mental status, and urinary retention following benztropine exposure. Although these limited signs may indicate autonomic instability, the patient did not exhibit all signs of anticholinergic toxicity such as mydriasis, dry skin/mucous membranes, or flushing, despite the electronic medical record documenting an anticholinergic toxicity diagnosis. This especially complicated the ability to discern between AIP and possible anticholinergic toxicity. The drooling present in the patient would likely lead away from a definitive anticholinergic toxicity diagnosis, but this could likely be attributed to the parkinsonian-like symptoms instead. It is still possible that anticholinergic side effects were co-occurring alongside AIP, rather than a single syndrome of AIP by itself. When benztropine was discontinued, the patient subsequently improved. Afterwards, amantadine was initiated as a short-term treatment strategy for EPS, which temporarily improved the patient’s symptoms after hospital admission. This clinical picture is clinically suspicious of anticholinergic effects of benztropine, but less strongly associated with a definitive classic anticholinergic toxidrome. 

This clinical scenario could be explained mechanistically by the complex interplay between dopaminergic and cholinergic pathways involved in motor control. illustrating the various receptors and neurotransmitters involved in the sequence of this patient’s clinical course. Dopaminergic signaling in the basal ganglia modulates motor control through a complex circuitry of excitatory and inhibitory signals with the direct and indirect pathways, which include the cerebral cortex, striatum, globus pallidus, thalamus, subthalamic nucleus, and substantia nigra. Antipsychotic medications, such as paliperidone, inhibit dopamine signaling in these circuits, disrupting motor regulation and modulation, producing parkinsonian symptoms (Figure 1). Discontinuation of benztropine may improve anticholinergic toxidrome, while amantadine may improve AIP through multiple proposed pharmacologic mechanisms, although the exact mechanisms of action are not fully understood (Figure 2) [7]. Amantadine has demonstrated benefits in AIP management and lacks anticholinergic properties, which makes it particularly effective as an alternative treatment for AIP when anticholinergic medications are not tolerated or their adverse effects are a concern [6]. 

**Figure 1:**
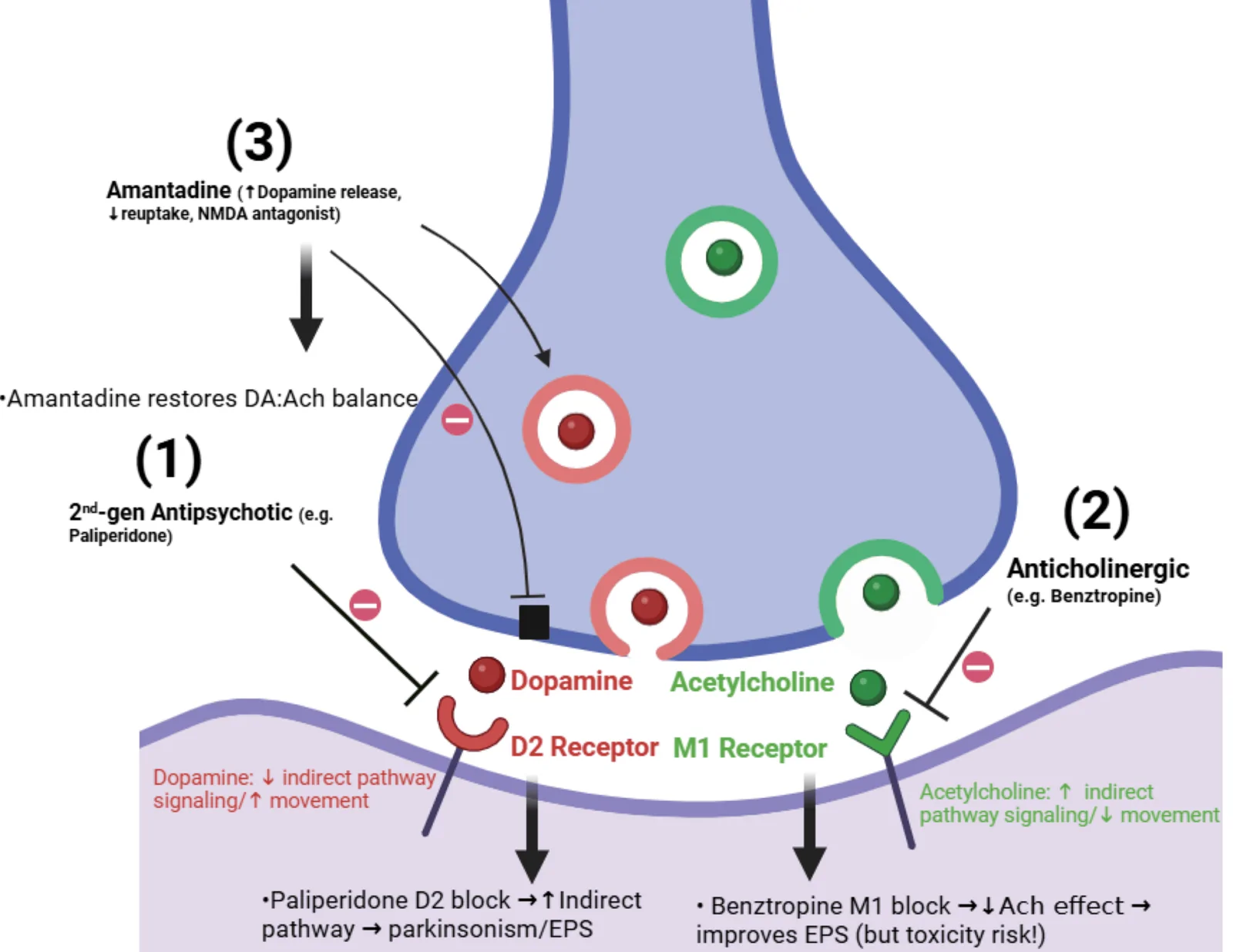
Dopamine-Acetylcholine Balance The interplay of dopaminergic and acetylcholinergic balance involved in motor control in this patient. Paliperidone blocks D2 dopamine receptors, upregulating the indirect pathway, leading to EPS. Benztropine blocks M1 acetylcholine receptors to restore dopamine balance, improving EPS, but associated with anticholinergic toxicity. Amantadine is proposed to act through multiple pharmacologic mechanisms including modulating dopamine release from synaptic vesicles, antagonizing NMDA receptors, and decreasing reuptake, which may treat AIP, although its exact mechanisms are not fully established. “Dopamine-Acetylcholine Balance” created in BioRender. Barnett, C. (https://BioRender.com/tqjd9kz) is licensed under CC BY 4.0. DA: dopamine; Ach: acetylcholine; NMDA: n-methyl-d-aspartate; EPS: extrapyramidal symptoms; D2: dopamine receptor 2; M1: muscarinic receptor 1; AIP: antipsychotic-induced parkinsonism

**Figure 2:**
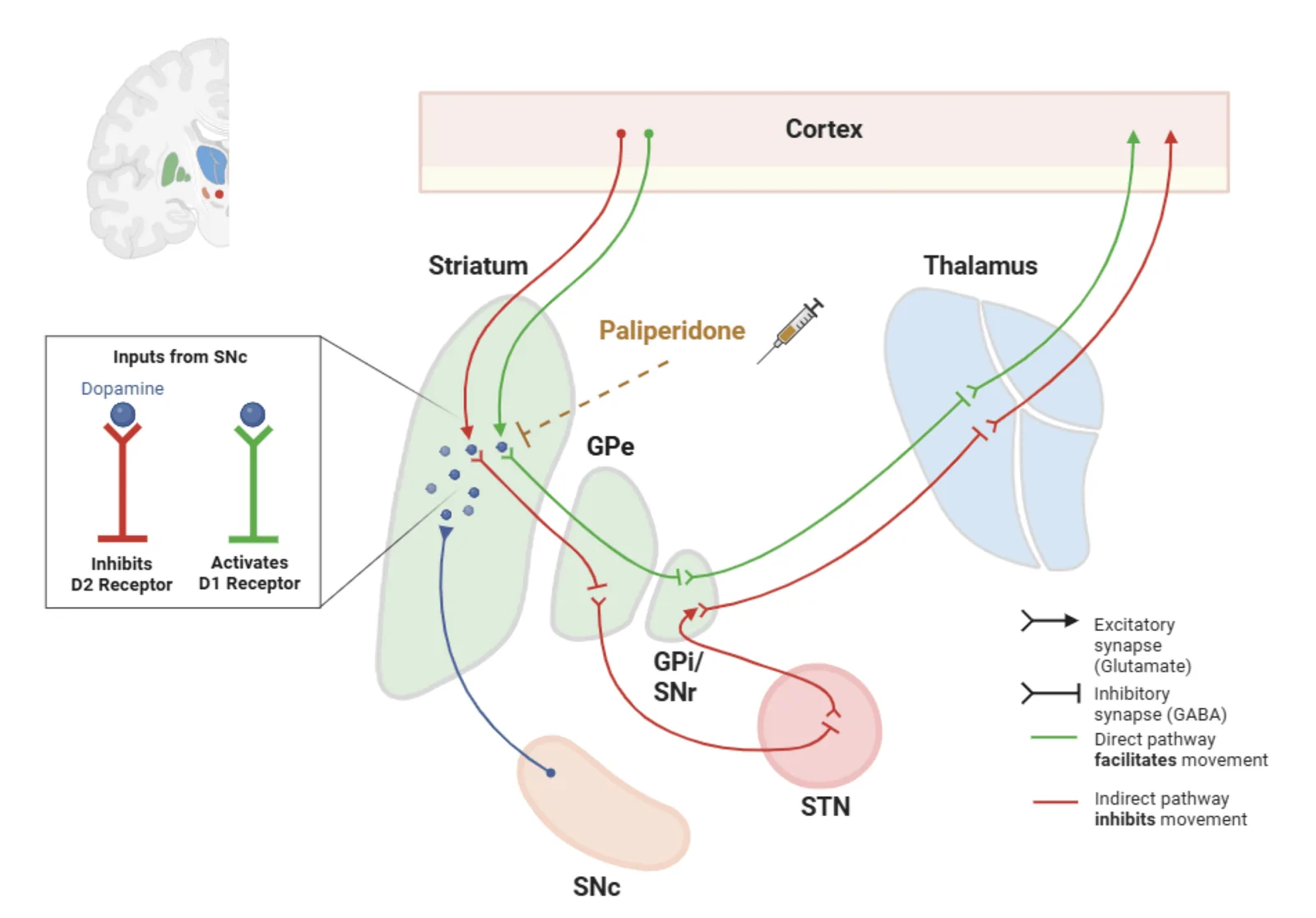
Dopaminergic Circuitry Mechanism of antipsychotic-induced parkinsonism/EPS within the basal ganglia. Dopamine released from the substantia nigra pars compacta (SNc) to the striatum modulates motor output through direct and indirect pathways. Paliperidone blocks D2 signaling and favors output from the indirect pathway, which ultimately suppresses thalamocortical control of motor activity and results in EPS. “Dopaminergic Circuitry” created in BioRender. Barnett, C. (https://BioRender.com/4t7050w) is licensed under CC BY 4.0. D2: dopamine receptor 2; D1: dopamine receptor 1; GPe: globus pallidus externus; GPi: globus pallidus internus; SNr: substantia nigra pars reticulata; STN: subthalamic nucleus; GABA: gamma-aminobutyric acid; EPS: extrapyramidal symptoms

Complex clinical presentations such as this can easily be mistaken for other toxidromes such as neuroleptic malignant syndrome (NMS), a life-threatening reaction to dopamine-blocking agents, characterized by hyperthermia, autonomic dysfunction, and altered mental status, which can lead to acute kidney injury and rhabdomyolysis [8]. Hyperthermia and lead-pipe rigidity, with a normal creatine kinase, make NMS less likely, but not a definitive exclusion, in this case. As such, the presence of anticholinergic toxicity may further confound the clinical picture to distinguish NMS from AIP with overlapping anticholinergic effects [9]. This autonomic instability in the absence of classic NMS findings (such as diaphoresis, elevated CK, hyperthermia, and lead-pipe rigidity) is, instead, likely due to benztropine anticholinergic side effects. Thus, it is essential for clinicians to recognize this diagnostic pitfall to avoid missing other life-threatening complications that may present similarly to AIP. 

This patient case also emphasizes the role of medication reconciliation in identifying and alleviating drug-induced syndromes. This patient presentation entailed close monitoring and intervention of AIP due to long-acting paliperidone injection for chronic schizophrenia management with subsequent anticholinergic toxic effects in an attempt to alleviate dopaminergic upregulation. 

Management included discontinuation of benztropine medication due to high initial suspicion for anticholinergic toxicity as noted in the medical records. Although benztropine was discontinued, the patient's parkinsonian symptoms still required management. As such, amantadine was initiated as an alternative to benztropine during transfer to higher levels of care at another facility for inpatient management. 

From a systems perspective, this case is particularly relevant to a community emergency department setting where limited access to subspecialists is available. Transfer of care between facilities is also highly important for continuous interventions in presentations such as this one. Furthermore, concurrent diagnoses related to AIP and anticholinergic side effects from medications such as benztropine must be carefully considered during transfer to higher levels of care. 

## Conclusions

This case emphasizes the diagnostic complexity of AIP in the setting of concurrent anticholinergic side effects from benztropine. Cases such as these still carry genuine diagnostic uncertainty and management challenges. Careful assessment of key distinguishing features of AIP with anticholinergic toxicity is essential to help clinicians recognize this clinical picture, avoid misdiagnosis, and provide appropriate treatment to prevent complications.
